# We Made Your Bed, Why Won’t You Lie in It? Food Availability and Disease May Affect Reproductive Output of Reintroduced Frogs

**DOI:** 10.1371/journal.pone.0159143

**Published:** 2016-07-27

**Authors:** Kaya Klop-Toker, Jose Valdez, Michelle Stockwell, Loren Fardell, Simon Clulow, John Clulow, Michael Mahony

**Affiliations:** Conservation Biology Research Group, School of Environmental and Life Science, University of Newcastle, Callaghan, 2308, NSW, Australia; Charles University in Prague, CZECH REPUBLIC

## Abstract

Mitigation to offset the impacts of land development is becoming increasingly common, with reintroductions and created habitat programs used as key actions. However, numerous reviews cite high rates of poor success from these programs, and a need for improved monitoring and scientific testing to evaluate outcomes and improve management actions. We conducted extensive monitoring of a released population of endangered green and golden bell frogs, *Litoria aurea*, within a created habitat, as well as complementary surveys of a surrounding wild population. We then compared differences between the created habitat and natural ponds where extant frogs either bred or didn’t breed in order to determine factors that contributed to the breeding failure within the created habitat. We evaluated differences of *L*. *aurea* abundance, abundance of other fauna, vegetation, water quality, habitat structure, invasive fish, and disease between the three pond types (created habitat, breeding ponds, non-breeding ponds). We discovered that vegetation and invertebrate diversity were low within the created habitat, potentially reducing energy and nutritional resources required for breeding. Also, a greater proportion of frogs in the created habitat were carrying the chytrid fungal pathogen, *Batrachochytrium dendrobatidis*, compared to the wild populations. In addition to causing the potentially fatal disease, chytridiomycosis, this pathogen has been shown to reduce reproductive functioning in male *L*. *aurea*, and subsequently may have reduced reproductive activities in the created habitat. Conspecific attraction, pond hydrology, and aquatic vegetation may also have had some influence on breeding behaviours, whilst the presence of the invasive mosquitofish, *Gambusia holbrooki*, and heterospecific tadpoles were unlikely to have deterred *L*. *aurea* from breeding within the created habitat. Through the use of scientific testing and monitoring, this study is able to make recommendations for future amphibian breed and release programs, and suggests planting a diversity of plant species to attract invertebrates, creating some permanent ponds, connecting habitat with existing populations, trialling artificial mating calls, and following recommendations to reduce the prevalence of disease within the population.

## Introduction

Anthropogenic habitat loss and degradation is the leading cause of global species decline [1]. In response, many countries have established legislative strategies centred on habitat offsetting and/or land mitigation to curb environmental damage and species loss [2, 3]. Offsetting commonly involves the restoration of degraded habitat or creation of a certain habitat type, to improve suitability for either a particular species or ecosystem biodiversity. Although, it is worth noting that in some cases, threatened species will occasionally inhabit disturbed habitats [4, 5]. When land is created for a particular species, translocations or captive breed and release programs are commonly associated [6, 7]. However, whilst these considerable conservation efforts are largely a positive step forward, there are many factors, including our limited understanding of the complex interactions within natural systems, that make these undertakings difficult, and success rare [8, 9].

Whilst the intuitive goal of species-level translocations or created habitat programs is to create a self-sustaining viable population of the target species, the release of captive bred animals alone, is not enough to ensure breeding success. This is because a variety of factors including nutrition, disease, habitat, and the density of individuals all play a large role in reproductive output. Reproduction is energetically costly, and physiological functions such as gonad growth and development, the growth and maintenance of secondary sexual features, and pursuing a mate, are typically only achieved by healthy individuals with an ample food supply [9, 10]. An adequate food intake can also increase reproductive success by helping individuals avoid disease or predation [11, 12]. Additionally, habitat must also contain appropriate nesting materials and oviposition sites, and provide food and shelter for progeny–whose requirements may be vastly different to that of adults [13]. The density of sexually mature individuals may also limit reproductive rates [14, 15]. Translocated populations are at particular risk of low density impacts because translocated animals are susceptible to high rates of predation and dispersal [6]. Therefore, whilst a created or restored habitat may look superficially the same as undisturbed habitat, there are a great number of factors required to support a self-sustaining population that may be difficult to identify without in-depth monitoring or experimentation.

Numerous reviews discussing the use of either translocations or created habitat suggest that increased monitoring and reporting of results, even unsuccessful ones, is vital for improving the efficiency and success of these high-level conservation programs [6, 7, 16,]. Several authors also encourage an increased use of scientifically testable monitoring within a framework of *a priori* questions [7, 17, 18,]. This kind of targeted monitoring can provide information on how released animals are using the habitat, mechanisms driving population decline or growth, and insight into the natural history of the species. In cases where reintroductions occur near extant populations of the target species, monitoring of these extant populations can be used as reference sites to strengthen tested hypotheses [18]. All of this targeted information can be used to identify habitat features or demographic parameters that can be manipulated to increase the chance of a successful mitigation program.

One species that has been the target of several habitat rehabilitation and breed and release programs for mitigation purposes, is the Australian green and golden bell frog, *Litoria aurea* [19, 20, 21, 22]. This frog has disappeared from over 90% of its historical range, and now occurs in disjunct populations along the coast of south eastern Australia, predominantly in areas of urban or industrial development [23, 24]. Despite the large amount of effort put into the recovery of this species, success rates for habitat creation and breed and release programs are low [19, 21, 22]. Due to the high failure rate of previous offset programs for this species, we established a trial created habitat close to an extant population that enabled us to test whether the habitat created would support a viable population. Whilst the created habitat supported the continued survival and growth of released *L*. *aurea*, three and a half years of intensive, weekly monitoring revealed no evidence of breeding on site and the sub-population cannot yet be classified as self-sustaining. A lack of breeding by released animals, is a common factor attributed to the failure of multiple reintroduction programs [25, 26]. In order to identify why our released *L*. *aurea* did not breed in the created habitat, our study compares how habitat variables, demography, and threatening processes such as the amphibian chytrid fungus, *Batrachochytrium dendrobatidis*, and predacious mosquitofish *Gambusia holbrooki*, varied between our trial habitat, and sites where breeding did and did not occur in the natural habitat of an extant *L*. *aurea* population.

## Methods

All work was conducted under National Parks and Wildlife Service scientific license number SL101409 and approved by The University of Newcastle Animal Care and Ethics Committee (ACEC number A-2011-137, and A-2010-145), with funding from BHP Billiton

### Study site

#### Kooragang Island

This study took place on Kooragang Island, NSW, Australia (32°50–54'S, 151° 42–47'E). Kooragang Island is located in the mouth of the Hunter River, and is divided north and south by train tracks that separate industrial and national park sides of the island. The industrial side of the island is heavily paved with large vegetated areas and contains at least 14 permanent, 5 semi-permanent, and 4 ephemeral water bodies in various states of natural appearance (figures of a subset of ponds are included in S1–S4 Figs). The national park side of the island is dominated by *kikuyu* fields, salt marsh, mangroves, cattle farmland, and contains at least 5 permanent, 12 semi-permanent, and 11 ephemeral water bodies. *Litoria aurea* is known to have existed on Kooragang Island since the 1970s, and this population is considered one of the last strong extant populations in Australia [27, 28].

#### Created habitat

**Fig 1:**
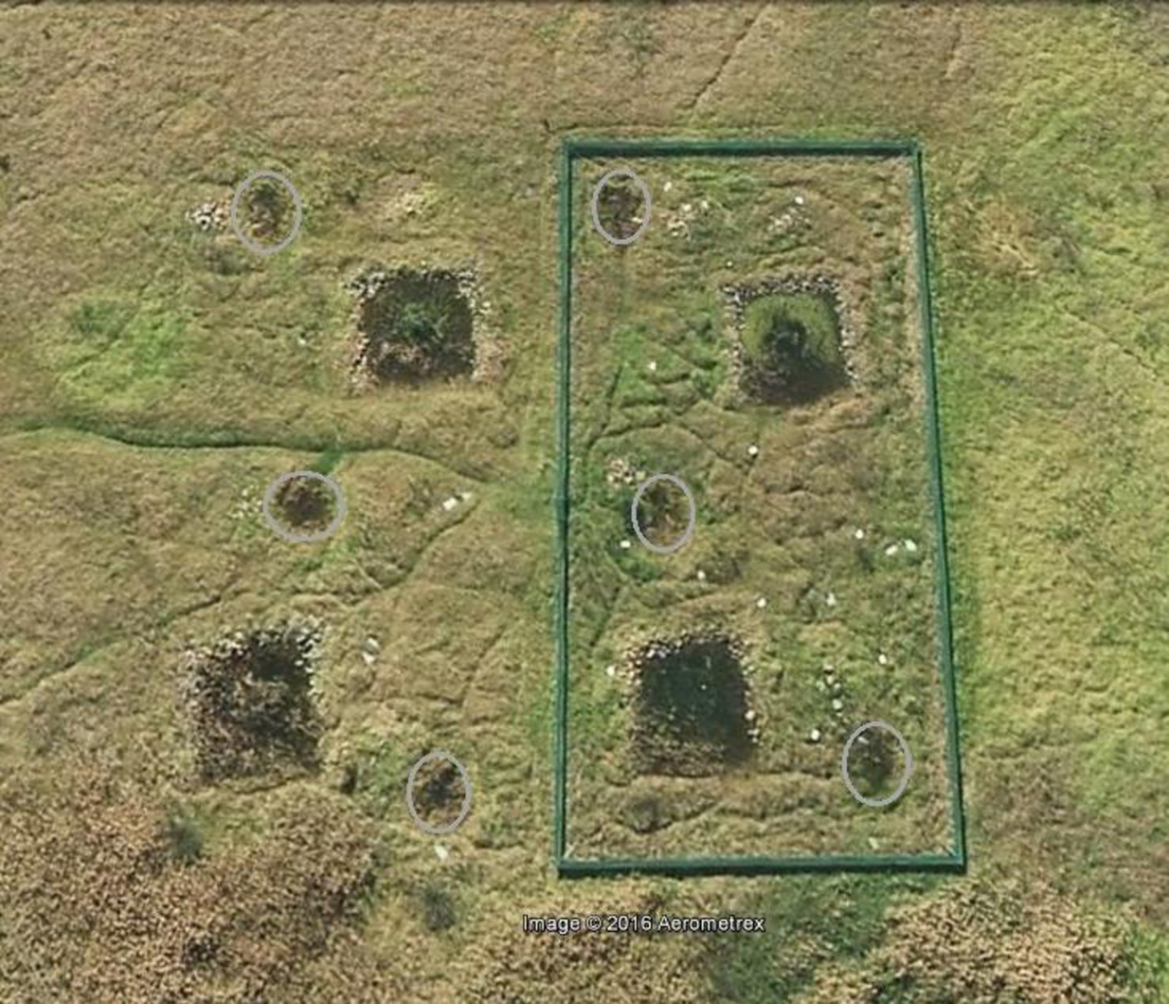
Satellite image of the created habitat. Image set at 100 m elevation. Large green rectangle is the frog and predator-proof fence. Square ponds are the permanent ponds. Ovals represent ephemeral ponds.

Situated within the national park on the southwest side of Kooragang Island, the created habitat consisted of ten constructed ponds; four square 64 m^2^ semi-permanent ponds approximately 1.5 m at the deepest point, and six smaller oval ephemeral ponds 8 m^2^ and 40 cm at the deepest point, along with a natural waterbody (NWL) 270 m^2^ located 50 m north west of the constructed ponds. The created ponds were divided into two replicated sections where one of these sections was enclosed in a frog and predator proof fence (see Fig 1). Artificial structures such as terracotta tiles, tin sheets, hay bales, and rock piles were included as supplementary terrestrial habitat (following recommendations from [29]). The surrounding habitat of the trial site is wet pasture dominated by *Kikuyu* grass. This site was chosen due to its proximity to a proposed, larger compensatory habitat creation project. No *L*. *aurea* were found at the trial site’s location in industry conducted pre-construction surveys.

### Captive breeding

*Litoria aurea* tadpoles were produced from a captive colony held at the University of Newcastle, using parental adults sourced at random from the population on Kooragang Island [21]. All breeding adults were swabbed and screened for Bd via Taqman qPCR analysis to ensure they were negative for Bd before entering the breeding colony. Tadpoles were also screened for Bd via mouth swabbing (described in [30]) and Taqman qPCR analysis, with at least 60 tadpoles from each breeding tank screened to confirm an absence of infection prior to release (assuming 95% confidence of detecting the presence of Bd in one animal if the fungus was present in 5% of animals [31, 32]). Methods for adult swabbing and qPCR analysis are described below under the sub-headings of Field Surveys, Created habitat, and Bd Screening, respectively.

Between March 2011 and January 2012, 10,012 captive bred *L*. *aurea* tadpoles were released over two occasions into the four semi-permanent ponds and NWL (1804 tadpoles released into the fenced area, 5208 into the unfenced area, and 3000 into NWL). Before release, a visible implant elastomer (VIE, Northwest Marine Technology, Shaw Island, WA, USA) was injected subcutaneously on the right side of the lower lateral ventral surface of the abdomen of each tadpole, with a unique colour used for each of the three release points (i.e. yellow implants for tadpoles released inside the fence, red implants for outside the fence, and green implants for NWL). These implants were conserved through metamorphosis and allowed us to see movement of animals across the site [33].

### Fauna surveys

#### Created habitat

Between April 2011 and April 2014, weekly frog and tadpole surveys were conducted at all of the constructed ponds and NWL. Surveys began at sundown during the warmer months of September to May, and during the day in winter, to follow *L*. *aurea’s* activity patterns. Standardised auditory surveys [34] were conducted at the beginning and end of survey periods to identify the number and location of calling *L*. *aurea* and other frog species. Standardised visual encounter surveys (VES) were conducted for all ponds and the surrounding terrestrial habitat, and were completed when the search area had been thoroughly searched once with no overlap [35, 36]. During each survey, the presence and abundance of all anuran species was recorded. Detected *L*. *aurea* were captured by hand with a thin, disposable plastic bag. The location within the created habitat, time of capture, and activity of the frog were recorded, as was the habitat structure the frog was using during time of capture. Any frogs that were observed but not captured had the same capture information recorded and were listed as missed animals. Because the same area was searched no more than once per survey period, there was little chance of re-counting missed frogs. Upon survey completion, captured frogs were weighed with spring Pesola scales, had their snout to vent length (SVL) measured with dial callipers, the presence and colour of VIE recorded, and their sex determined by the presence or absence of nuptial pads for animals over 45 mm SVL, with those smaller than 45 mm recorded as juveniles [37]. Each frog was swabbed in a standardized manner (eight strokes to each dorsal-ventral surface, eight strokes to each inner thigh, and four to each of the hands and feet (modified from [38]) with a dry swab (Medical Wire and Equipment Company, Corsham, UK) to determine the presence of the fungal pathogen, *Batrachochytrium dendrobatidis* (Bd). Frogs were then scanned for the presence of a passive integrated transponder (PIT) tag inside the dorsal lymph space, using a Trovan LID-560ISP reader (Trovan Ltd., UK). Each PIT tag carries a unique code, recorded at each capture. If the frog was a new capture and > 35 mm SVL, a PIT tag was injected subcutaneously [39], and the unique number recorded. After processing, frogs were returned to their point of capture.

Dip-netting of every pond was conducted to identify breeding events through the presence of tadpoles, and to sample fish and aquatic invertebrate presence and abundance. Using fine meshed dip-nets with a triangular mouth—area of approximately 600cm^2^, semi-permanent ponds were swept ten times, ephemeral ponds swept five times, and NWL was swept 20 times. After sweeping, nets were inspected, and the presence and abundance of all tadpoles, fish and invertebrates were recorded. Tadpoles were then released back to their pond of capture.

#### Extant population

Four sets of VES and tadpole surveys were conducted each year at all of the known waterbodies on Kooragang Island (n = 58) during *L*. *aurea’s* breeding season (September to April) for the years 2011/2012, 2012/2013, and 2013/2014. VES at the extant ponds were very similar to VES at the created habitat, with searches being preceded by auditory surveys and water quality parameter collection. The perimeter of each pond, including a 1 m wide section of terrestrial habitat, was searched once, with all *L*. *aurea* collected or recorded as misses. The same habitat and frog morphometric data was recorded as in the created habitat with the addition of a GPS position for each frog captured.

Due to the large size of ponds on Kooragang Island, aquatic funnel traps (dimensions: 23x23x43 cm) were used to survey tadpoles, fish, and aquatic invertebrates. Thirteen cm activated fluorescent yellow glow-sticks, and 8–10 3mm Aquafeed trout pellets were placed in each trap to attract animals [40]. Traps were tied to emergent vegetation at the edge of ponds, approximately 10 m apart, with at least a third of the trap above water to provide air to captured animals. Traps were left overnight for 12 hours and inspected the following morning. All animals captured were recorded to species level, before being released at their point of capture. A breeding pond was defined as any pond where *L*. *aurea* tadpoles had been captured during that particular breeding season.

#### Invertebrates

Terrestrial invertebrate sampling was conducted ad-hoc to test the assumption that terrestrial invertebrate abundance is affected by vegetation diversity and differs between sites. Eighteen plastic pitfall traps (dimensions; 10 cm diameter, 4 cm deep) were set up in transects along a low vegetation (vegetation height <50cm) and high vegetation (vegetation height >2 m) zone at both the created habitat and a breeding pond (five traps at low vegetation zones, 4 traps at high vegetation zones). Traps were placed on the ground at a distance of >2 m apart and half filled with tap water and several drops of detergent to prevent invertebrates escaping. Trapping was conducted at the beginning of the 2015 breeding season for three nights over a two week period, totalling 54 trap nights. Traps were checked after 24 hours and all invertebrates >2 mm long were collected and recorded to either order or family.

### Habitat surveys

Before VES, water quality parameters including water temperature, salinity, pH, dissolved oxygen levels (DO), and conductivity were obtained with a YSI Professional Plus (Xylem, USA) water quality probe.

Habitat attributes were scored by estimating the percentage of habitat structures such as rocks and vegetation types, at each of the three pond sections: inside the pond, in a 2 m perimeter around the shoreline of a pond, and in a 20 m perimeter of riparian zone outside the pond, for all of the Kooragang Island natural ponds and created habitat ponds.

### Bd screening

DNA material was collected from swab tips by extraction with PrepMan Ultra. The tip of each swab was added to 50 μl of PrepMan Ultra and 30 to 40 mg of Zirconium/silica beads measuring 0.5 mm diameter (Biospec Products). Samples were homogenised for 45 seconds in a mini beadbeater (Biospec Products), then centrifuged at 2000 rpm for 2 minutes in a Centurion Scientific C2 series centrifuge. Homogenisation and spinning were repeated once more. The homogenised swab tip was then immersed in a water bath set at 100°C for 10 minutes, cooled for two minutes, and then re-centrifuged for three minutes to recover all material to the bottom of the tube. Five μl of this supernatant was added to 45 μl of Milli-Q water and frozen at -80°C.

The quantification of Bd on each swab was determined using the TaqMan qPCR method as previously reported [41] using a Rotor Gene 6000 (Corbett Life Science). The sequences of the primers and probe are as follows: 5′-CCTTGATATAATACAGTGTGCCATATGTC-3′ (forward primer), 5′-AGCCAAGAGATCCGTTGTCAAA-3′ (reverse primer), 5′- 6FAM CGAGTCGAACAAAAT MGBNFQ-3′ (FAM-labelled TaqMan probe). To verify the specificity of the primer/probe set, we compared their sequences against other biological sequences available in the Genbank database using the Basic Local Alignment Search Tool (BLAST) program. The search resulted in a total of 57 hits, which all belong to the 5.8S rRNA-internal transcribed spacer region isolated from different strains of *B*. *dendrobatidis*, supporting the amplification specificity of the primer/probe set. A 1.5-μl aliquot of TaqMan Exogenous Internal Positive Control Reagents (Applied Biosystems), containing 0.5 μl exogenous positive control DNA and 1 μl pre-designed primers and VIC-labelled TaqMan probe (commercial sequences not provided by the manufacturer), was spiked into each sample. This positive control serves the purpose to distinguish true target negatives from false target negatives due to PCR inhibition. In the qPCR, the target and the positive control are co-amplified and detected by FAM and VIC fluorescence, respectively. The amplification results were interpreted as follows: (1) a negative signal (C_T_ ≥ 40) for the target and a positive signal (C_T_ ≤ 40) for the positive control indicate the absence of the target (i.e., a true target negative); (2) negative signals for both the target and the positive control suggest a false target negative (due to PCR inhibition). Samples from false negatives were diluted with sterile Milli-Q water to a 1/100 solution to reduce the concentration of PCR inhibitors before they were re-analysed by qPCR. A calibration standard with a known concentration of Bd DNA (Pisces Molecular Llc) was diluted to give 0.1, 1, 10 and 100 Bd genomic equivalents (i.e., the quantities of DNA present in 0.1, 1, 10 and 100 Bd zoospores, respectively). This DNA dilution series was included in each qPCR run to generate a standard curve from which the quantity of Bd DNA in samples could be determined. For all samples, a geometric mean was calculated from the three replicates (including equivocal results where at least one of the replicates returned a zero value) for the quantification purpose. Finally, Bd zoospore equivalents are calculated by multiplying the genomic equivalent values by an appropriate dilution factor which accounts for the dilution of samples made in the PCR (i.e., 10 for normal samples or 100 for samples initially determined to be false negative). Samples showing zoospore equivalents ≥1 were considered infected (i.e., Bd-positive) whereas those with zoospore equivalents <1 were considered uninfected (i.e., Bd-negative).

In order to detect Bd with 95% confidence within each pond place, ponds that contained a high abundance of *L*. *aurea* (>60 frogs [32]), and were of a comparable area to the created habitat, were selected for additional surveying and Bd screening. At the two high-abundance ponds selected (one a breeding pond (S1 Fig), one a non-breeding pond (S3 Fig)), capture-mark-recapture (CMR) surveys followed immediately after the initial VES. Frog capture and data collection procedures were conserved for both survey techniques, with the only difference being the length of search time: CMR surveys continued until no more frogs were observed by searchers during two consecutive perimeter searches. CMR surveys were also repeated the following night for up to 7 consecutive nights, or until a 60% recapture rate was achieved. These additional surveys only took place during the first and last survey period of each season because Bd is no longer detectable in this population during the middle of summer, likely due to the ambient temperature exceeding the survivable tolerance limits for this pathogen [38].

### Statistical analysis

A main dataset was built by combining survey data from pre-existing Kooragang Island ponds with survey data from the created habitat. Created habitat data used in this combined data set was taken from surveys conducted the same week as Kooragang Island surveys. The variable of “pond place” was created to denote if a pond was from the created habitat (CH), or if it was a pre-existing Kooragang Island breeding pond (BP) or non-breeding pond (NBP) for *L*. *aurea*.

#### Habitat

To compare differences between the created habitat, breeding ponds, and non-breeding ponds (pond place) a one-way analysis of variance (ANOVA) was conducted on the means of abiotic parameters including water quality, pond characteristics, and percent of vegetation types at each pond place. This analysis was also conducted on aquatic invertebrate abundance and diversity at the created habitat, to compare differences between the inside and outside of the fenced section in the created habitat. Analysis of vegetation types was segregated between the three vegetation zones; riparian, shoreline, and emergent. When ANOVAs were significant, they were followed with a Student’s t-test to test for differences between the means of each site. Where the data were not normally distributed, a Kruskal-Wallis nonparametric test was also used. When this test was significant it was followed with a Wilcoxon each pair test to identify differences between the means at each site. These models were also used to analyse differences in terrestrial invertebrate abundance and diversity for high and low vegetation zones at the created habitat and breeding pond.

#### Threats

To compare differences in the presence of *G*. *holbrooki* between pond places, chi squared analysis was used. The variable of *G*. *holbrooki* presence was created for each pond by scoring ponds with a “0” if *G*. *holbrooki* had never been observed, or a “1” if *G*. *holbrooki* had been observed in that pond during at least one survey period.

Chi squared analysis was used to compare differences in Bd prevalence (i.e. the proportion of frogs infected) between the created habitat and the pond places where CMR was conducted. A one-way ANOVA was used to compare differences in the severity of infected *L*. *aurea* between these sites. Only the first observation of each frog within a weekly survey period was used, with secondary observations removed prior to analysis to prevent pseudo replication.

All of the above modelling was conducted in JMP v11.

#### Fauna

Due to the possibility of zero inflation in fauna abundance data, the means of these parameters (i.e. total, male, female, juvenile *L*. *aurea* abundance, and abundance of heterospecific frogs at each pond) were analysed against pond place using generalized linear models with a log link function in the program SAS v 9.3. These models were built with just the Poisson distribution (P) when no overdispersion was found, otherwise a series of additional models were built with the following distributions: zero inflated Poisson (ZIP); negative binomial (NB); and zero inflated negative binomial (ZINB), to test if the overdispersion was due to just zero inflation, excess Poisson variability or a combination of both respectively. Model significance tests, the Akaike Information Criterion (AIC), and the amount of overdispersion were used to determine the best model, given in the results. When the top model was selected, we used differences in least squares means to determine the differences between means for each “pond place” each survey year, and differences between years for each pond place.

## Results

### Fauna abundance

#### *Litoria aurea* demography

**Fig 2:**
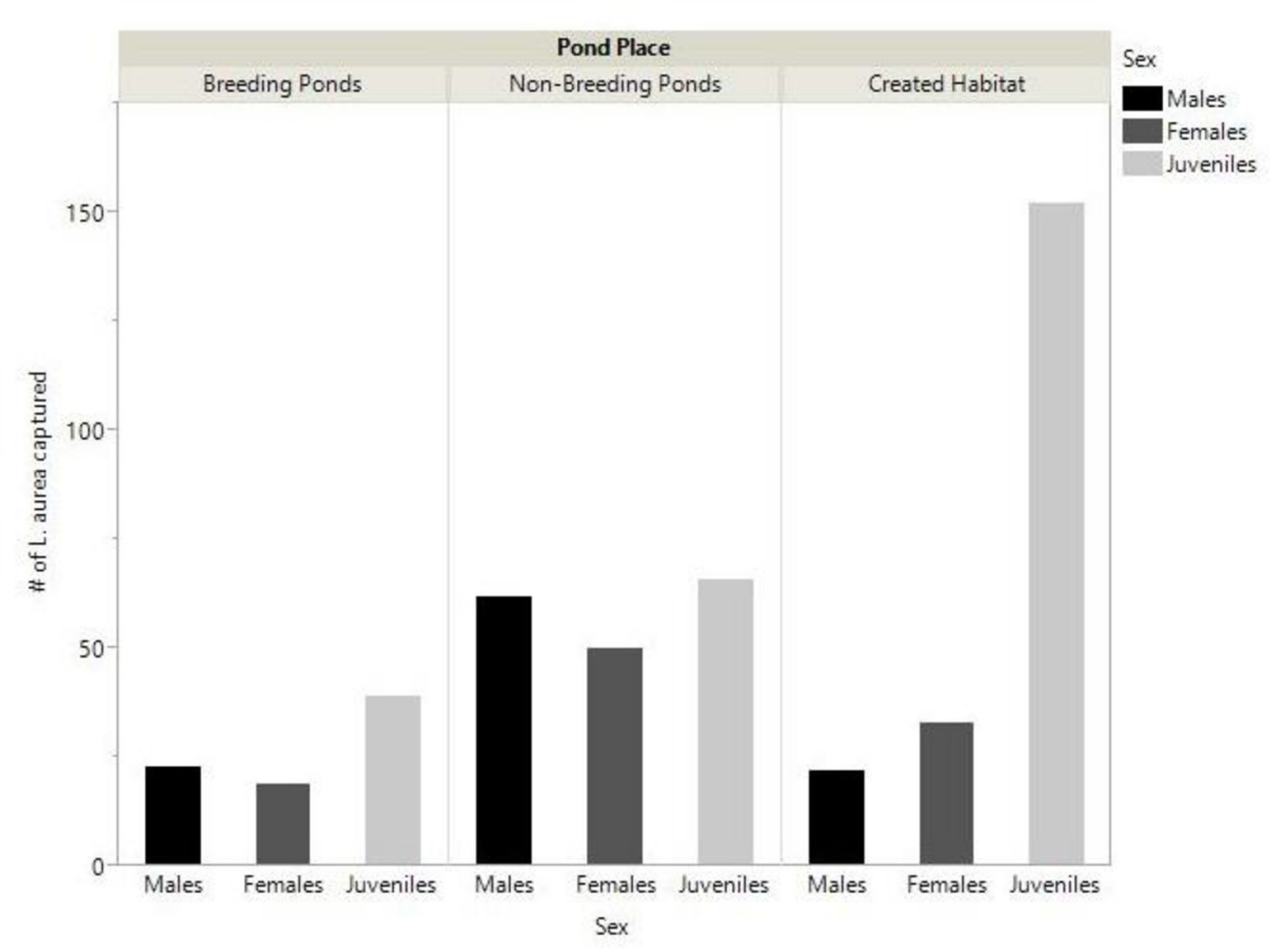
Abundance of captured *L*. *aurea*. Total number of individually marked *L*. *aurea* captured at each of the three pond places, separated by sex.

During the three years of VES surveys, a combined total of 572 frogs were encountered across all the surveyed ponds, with 471 of these frogs captured and processed. Of the 471 captures, 81 were from the breeding ponds, 178 from the non-breeding ponds, with 207 from the created habitat. Missed frogs were fairly consistent across sites, with 27 missed at the breeding ponds, 42 at the non-breeding ponds, and 32 at the created habitat. At both the breeding and non-breeding ponds, males outnumbered females by approximately 20%. This trend was reversed in the created habitat, where females outnumbered males by approximately 30% (Fig 2).

**Fig 3:**
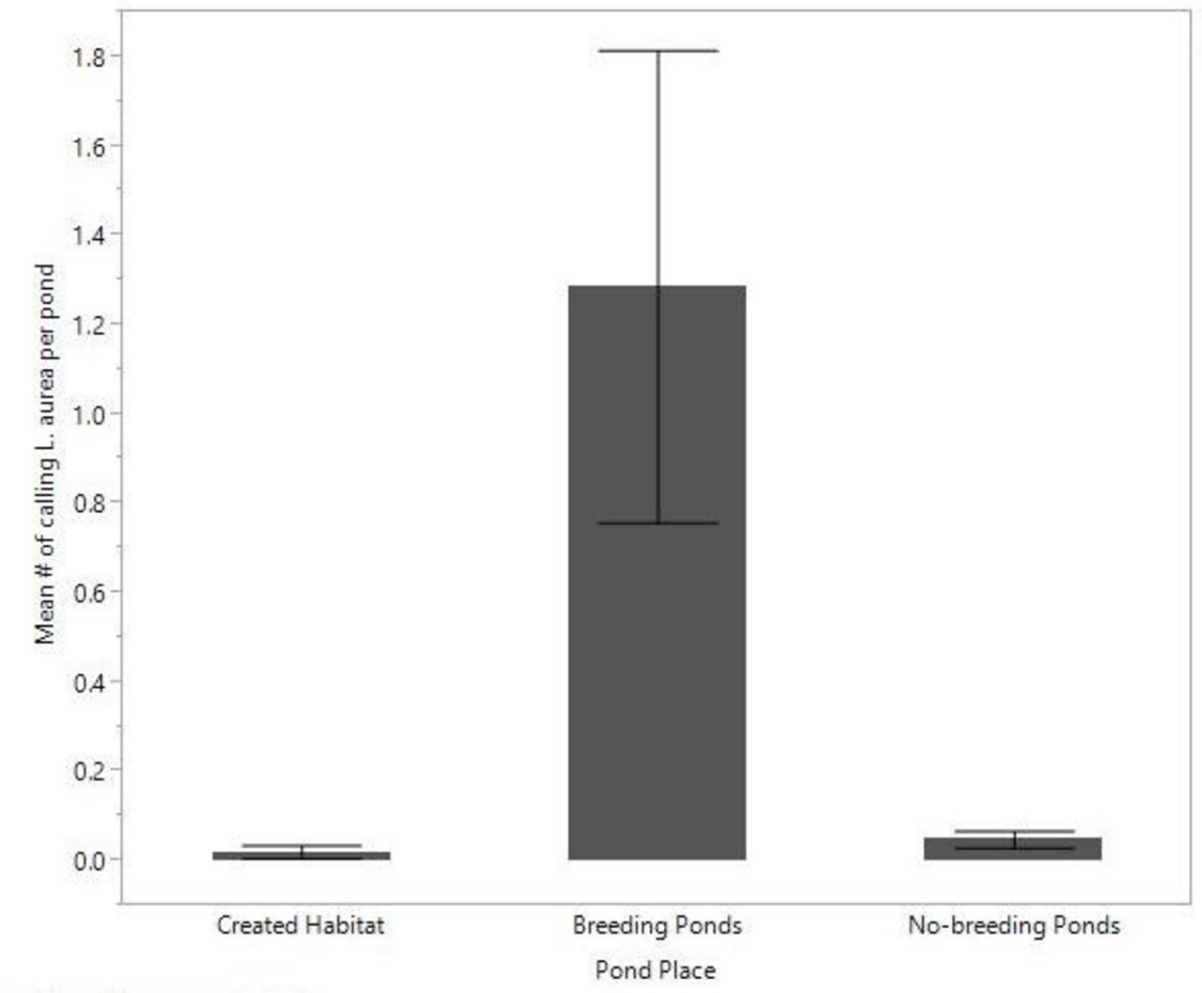
Mean number of *L*. *aurea* heard calling per pond across each pond place. Error bars represent 95% confidence intervals.

The generalised linear models, with a ZINB distribution, showed that each year, breeding ponds had the highest mean abundance of *L*. *aurea*, including both reproductively mature males and females (Table 1). However, these abundances were only significantly greater than those in the created habitat in year two for males and year three for females, and were never significantly greater than abundances at the non-breeding ponds (Tables 1 & 2). The abundances of male and female *L*. *aurea* did not differ significantly between years, with the exception of fewer females in the created habitat in the second year compared to the first year (Table 2). There were also significantly more calling males at breeding ponds compared to both the created habitat (*Z* = 4.9, *P* < 0.001) and non-breeding ponds (*Z* = 5.5, *P* = < 0.001) (Fig 3).

**Table 1:** Mean abundance of *L*. *aurea* females and males per pond at each pond place for the three survey seasons. SEM = standard error of the mean

	Created Habitat		Breeding Ponds		Non-Breeding Ponds	
Year	Mean	SEM	Mean	SEM	Mean	SEM
**Females**						
1	0.58	0.16	2.7	3.27	0.58	0.18
2	0.21	0.09	0.54	0.51	0.76	0.25
3	0.022	0.09	1.65	1.35	1.09	0.46
**Males**						
1	0.27	0.11	1.66	2.15	0.51	0.35
2	0.15	0.08	1.5	1.46	1.07	0.63
3	0.26	0.11	2.58	3.46	2.14	1.12

**Table 2:** Results from the generalised linear model with a zero inflated negative binomial distribution showing differences in male and female *L*. *aurea* abundance estimates between years and between sites with corresponding test statistics and significance values. Significant results are denoted with bold font. CH = Created Habitat; BP = Breeding Ponds; NBP = Non-Breeding Ponds

Females								
Pond Place	Survey year	Pond Place	Survey year	Estimate	Lower CI	Upper CI	Z	*P*
**CH**	**1**	**CH**	**2**	**2.71**	**1.02**	**7.20**	**2.00**	**0.05**
CH	1	BP	1	0.21	0.02	2.44	-1.24	0.21
CH	1	NBP	1	0.99	0.27	3.63	-0.01	0.99
CH	2	CH	3	0.94	0.30	2.95	-0.11	0.91
CH	2	BP	2	0.39	0.05	2.86	-0.93	0.35
CH	2	NBP	2	0.28	0.07	1.09	-1.84	0.07
**CH**	**3**	**BP**	**3**	**0.14**	**0.02**	**0.83**	**-2.17**	**0.03**
**CH**	**3**	**NBP**	**3**	**0.21**	**0.06**	**0.68**	**-2.61**	**0.01**
BP	1	BP	2	4.96	0.30	81.26	1.12	0.26
BP	1	NBP	1	4.65	0.45	48.30	1.29	0.20
BP	2	BP	3	0.33	0.03	3.58	-0.91	0.36
BP	2	NBP	2	0.71	0.09	5.38	-0.33	0.74
BP	3	NBP	3	1.52	0.26	8.86	0.46	0.64
NBP	1	NBP	2	0.76	0.18	3.14	-0.38	0.71
NBP	2	NBP	3	0.70	0.21	2.34	-0.58	0.57
**MALES**								
CH	1	CH	2	1.80	0.50	6.43	0.9	0.37
CH	1	BP	1	0.16	0.01	2.38	1.32	0.19
CH	1	NBP	1	0.54	0.11	2.64	-0.76	0.45
CH	2	CH	3	0.59	0.16	2.15	-0.8	0.42
**CH**	**2**	**BP**	**2**	**0.10**	**0.01**	**0.87**	**-2.09**	**0.04**
**CH**	**2**	**NBP**	**2**	**0.14**	**0.03**	**0.65**	**-2.52**	**0.01**
CH	3	BP	3	0.10	0.01	1.59	-1.63	0.10
**CH**	**3**	**NBP**	**3**	**0.12**	**0.03**	**0.45**	**-3.13**	**0.002**
BP	1	BP	2	1.11	0.06	20.41	0.07	0.95
BP	1	NBP	1	3.28	0.26	41.33	0.92	0.36
BP	2	BP	3	0.58	0.03	12.72	-0.34	0.73
BP	2	NBP	2	1.40	0.18	11.08	0.32	0.75
BP	3	NBP	3	1.20	0.08	17.91	0.13	0.89
NBP	1	NBP	2	0.47	0.10	2.14	-0.98	0.33
NBP	2	NBP	3	0.50	0.14	1.82	-1.05	0.29

*Litoria aurea* tadpoles were captured in eight of the 58 natural ponds at least once during the period of the study. However, the presence of *L*. *aurea* tadpoles were never detected in the created habitat. Despite the lack of tadpoles at the created habitat, juvenile *L*. *aurea* were detected in 2013 that were too small to have been captive-bred and released animals and did not have a VIE tag. Relatedness estimates from genetic analysis revealed that there is a high probability that a proportion of these juveniles were from at least one clutch parented by a released *L*. *aurea* (Valdez et al. 2015 unpublished data).

#### Other anurans

Breeding by at least one other frog species (including *Crinia signifera*, *Litoria dentata*, *Litoria fallax*, *Litoria peronii*, or *Limnodynastes peronii*,) occurred in every created pond. The abundance of heterospecific tadpoles at the created habitat did not differ from breeding ponds (*Z* = 1.18, *P* = 0.24) or non-breeding ponds (*Z* = 1.43, *P* = 0.15). However, the abundance of these tadpoles at breeding ponds was significantly higher than abundances encountered at non-breeding ponds (*Z* = 2.21, *P* = 0.03).

#### Invertebrates

**Fig 4:**
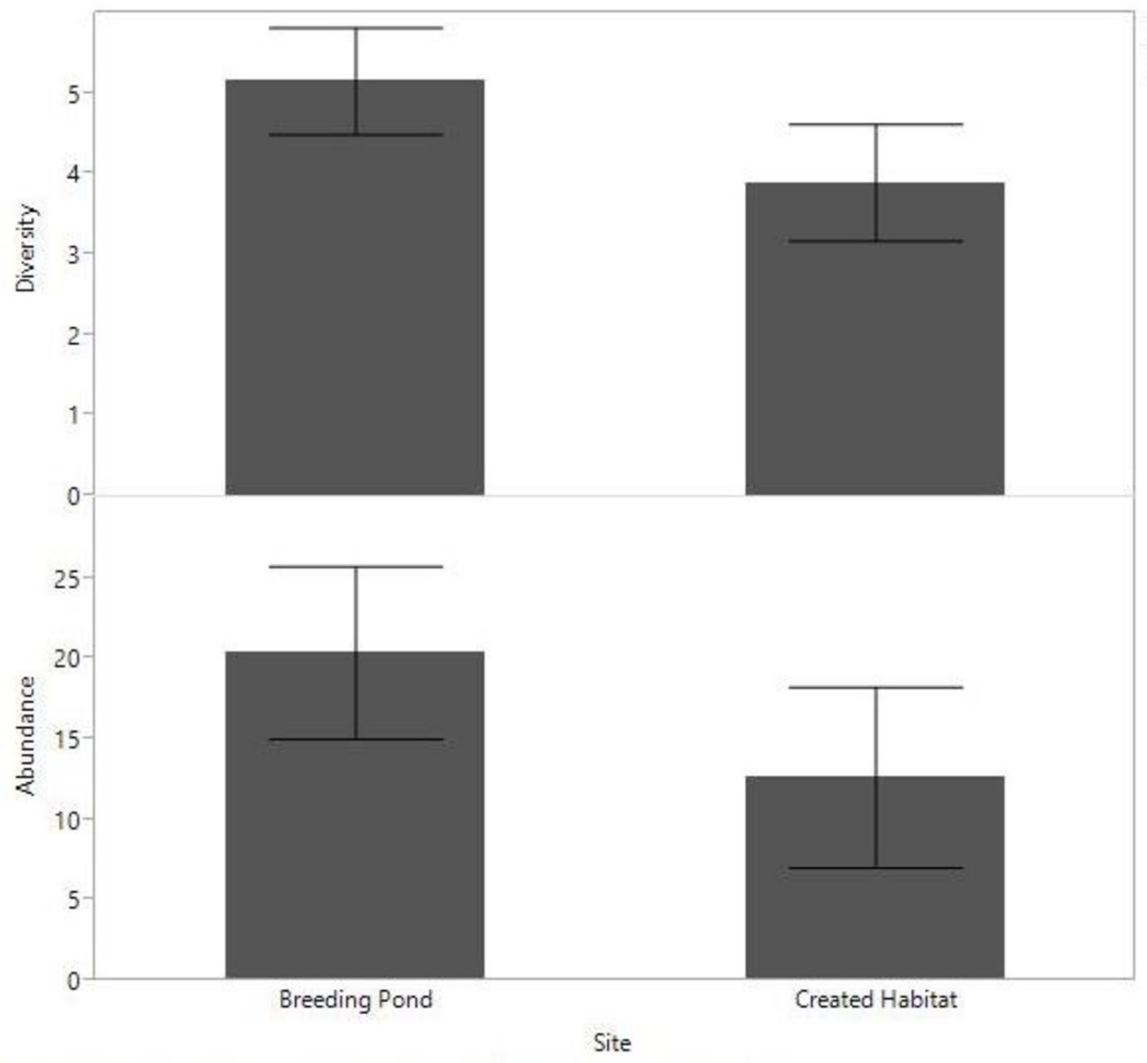
Mean abundance and diversity of terrestrial invertebrates captured in pit-fall traps at the created habitat and breeding pond. Error bars represent 95% confidence intervals

The mean abundance (mean ± SD, 20.4 ± 13.3) and diversity (5.2 ± 1.6) of invertebrates per trap at the breeding pond, was significantly greater than the abundance (12.6 ± 14.5) and diversity (3.9 ± 1.9) at the created habitat (*Z =* 3.3, *P <* 0.001, *Z* = 2.4, *P* = 0.02, respectively) (Fig 4). Invertebrate diversity was significantly higher in the low vegetation zone at the breeding pond compared to the high vegetation zone (*Z* = 2.4, *P* = 0.014). This trend was reversed in the created habitat with high diversity in the high vegetation, but this was not statistically significant (*Z* = 1.6, *P* = 0.09). There was also no difference between invertebrate abundance between high and low vegetation zones at either the created habitat (*Z* = 1.1, *P* = 0.25), or breeding pond (*Z* = 1.9, *P* = 0.051).

### Habitat structures and vegetation

#### Riparian zone

The greatest habitat differences between the created habitat and breeding ponds were seen in the riparian zone (Table 3). The non-parametric tests showed that compared to created habitat, breeding and non-breeding ponds both had significantly higher proportions of riparian trees (both; *Z* > 8.2, *P* < 0.001), bushes (both; *Z* > 3.5, *P* < 0.001), the common reed, *Phragmites australis* (BP; *Z* = 4.3, *P* < 0.001, NBP; *Z* = 2.7, *P* = 0.007), and the introduced spikey rush, *Juncus acutus* (BP; *Z* = 5.7, NBP; *Z* = 6.1, both; *P* < 0.001). Whereas the created habitat contained significantly greater proportions of grass (BP; *Z* = 4.6, NBP; *Z* = 11.4, both; *P* < 0.001), and rocks (BP; *Z* = 2.0, *P* = 0.042, NBP; *Z* = 10.6, *P* < 0.001). The created habitat also contained significantly less bulrush (*Typha orientalis*) compared to the non-breeding ponds (*Z* = 2, *P* = 0.04). There was no difference in the proportion of these habitat features between the breeding and non-breeding ponds, except for *P*. *australis*, which was significantly more abundant surrounding breeding ponds (*Z* = 3.3, *P* = 0.001), and grass which was significantly more abundant surrounding non-breeding ponds (*Z* = 3.1, *P* = 0.002) (Table 3).

**Table 3:** Mean (95% CI) proportions of vegetation types at each habitat site and vegetation zone.

		Mean proportion [95% CI]		
Zone	Plant	Created habitat	Breeding ponds	Non-breeding ponds
**Riparian**	**Grass**	81.1 [77.4, 84.8]^a^	13.b3 [7.0, 33.7]^b^	40.0 [36.8, 43.3]^c^
	**Trees**	0 ^a^	6.7 [0.6, 12.7] ^b^	16.4 [13.3, 19.4] ^b^
	**Bushes**	0.16 [0.05, 0.28] ^a^	3.9 [0.15, 7.6] ^b^	4.0 [3.0, 5.0] ^b^
	***T*. *orientalis***	4.1 [2.2, 6.0] ^a^	1.1 [-1.5, 3.7]^ab^	10.9 [8.3, 13.4]^b^
	***P*. *australis***	6.3 [3.9, 8.6] ^a^	25.0 [16.6, 33.4] ^b^	9.8 [7.9, 11.8]^c^
	***J*. *acutas***	0 ^a^	1.7 [-0.25, 3.6] ^b^	4.9 [3.8, 6.0] ^b^
**Shoreline**	**Grass**	42.6 [34.8, 50.5]^a^	1.7 [0.26, 3.6]^b^	29.8 [26.4, 33.4]^c^
	***T*. *orientalis***	6.9 [4.6, 9.1]^a^	11.1 [1.9, 24.1]^ab^	21.5 [18.3, 24.7]^b^
	***P*. *australis***	4.4 [2.2, 6.6]^a^	41.7 [33.5, 49.8]^b^	22.2 [18.5, 25.9]^c^
	***J*. *acutas***	0^a^	0^a^	9.7 [7.4, 12.0]^b^
	***Schoenoplectus***	1.9 [0.3, 3.4]^a^	20.0 [3.1, 43.1]^b^	2.6 [1.3, 4.0]^a^
	**Rock**	27.6 [21.1, 34.2]^a^	16.4 [0.3, 32.6]^a^	2.1 [1.2, 3.0]^b^
**Emergent**	**Grass**	17.3 [12.0, 22.6]^a^	8.3 [1.3, 17.9]^a^	14.3 [10.8, 17.9]^b^
	**Open water**	63.8 [58.7, 68.9]^a^	40.6 [17.0, 64.1]^b^	44.3 [39.4, 49.2]^b^
	**Floating**	4.8 [1.6, 8.0]^a^	28.3 [1.0, 55.6]^b^	26.1 [21.5, 30.8]^b^

Subscript letters denote significant differences between sites.

#### Shoreline

Similarly to the riparian habitat, shoreline vegetation of created ponds differed from both breeding and non-breeding ponds by having significantly higher proportions of grass (*Z* = 4.0, *P* < 0.001) and *P*. *australis* (*Z* = 5.8, *P* < 0.001). The percentage of sedges (*Schoenoplectus* spp.) was also lower than in breeding ponds (*Z* = 3.0, *P* = 0.003), but there was no difference to non-breeding ponds (*Z* = 0.99, *P* = 0.3). Created ponds also had lower proportions of trees (*Z* = 5.1, *P* < 0.001), the bulrush, *T*. *orientalis* (*Z* = 5.5, *P* < 0.001), and *J*. *acutus* (*Z* = 7.1, *P* < 0.001) along the shoreline compared to non-breeding ponds, and higher proportions of rocks (*Z* = 9.2, *P* < 0.001). The shoreline of breeding ponds also differed from non-breeding ponds by having significantly higher proportions of *P*. *australis* (*Z* = 2.9, *P* = 0.035), *Schoenoplectus* spp. (*Z* = 2.4, *P* = 0.015), and rock (*Z* = 4.5, *P* < 0.001), and significantly lower proportions of grass (*Z* = 3.2, *P* = 0.001) and *J*. *acutus* (*Z* = 2.3, *P* = 0.02) (Table 3).

#### Emergent

Ponds within the created habitat had a significantly higher percentage of open water compared to both breeding ponds (*Z* = 2.3, *P* = 0.02) and non-breeding ponds (*Z* = 3.8, *P* < 0.001), as well as a higher percentage of emergent aquatic grass inside the pond compared to non-breeding ponds (*Z* = 2.2, *P* = 0.026), but not the breeding ponds (*Z* = 0.5, *P* = 0.61). The inside of ponds within the created habitat had a lower mean percentage of floating vegetation (including the pond weed *Azolla*) compared to both the breeding and non-breeding ponds (BP; *Z* = 4.2, NBP; *Z* = 6.7, both; *P* < 0.001), and *P*. *australis* compared to non-breeding ponds (*Z* = 3.5, *P* < 0.001), but not breeding ponds (*Z* = 0.5, *P* = 0.62). The insides of both the created habitat and breeding ponds did not contain any *J*. *acutus*, *Schoenoplectus* spp, or trees either dead or alive, whereas each of these vegetation types occurred in at least one of the non-breeding ponds (Table 3).

#### Water quality

**Fig 5:**
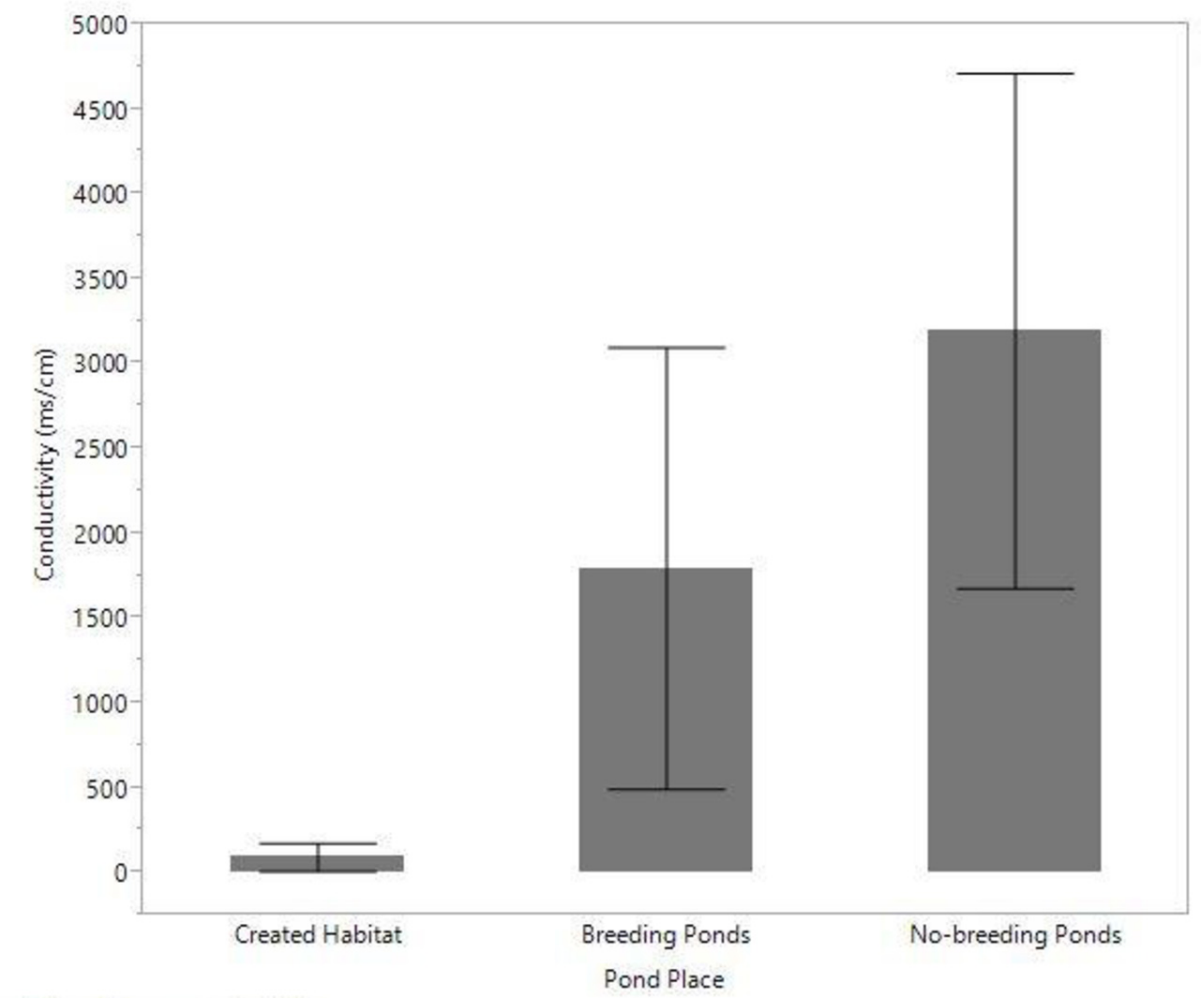
Mean pond conductivity across each of the pond places. Error bars represent 95% confidence intervals.

**Fig 6:**
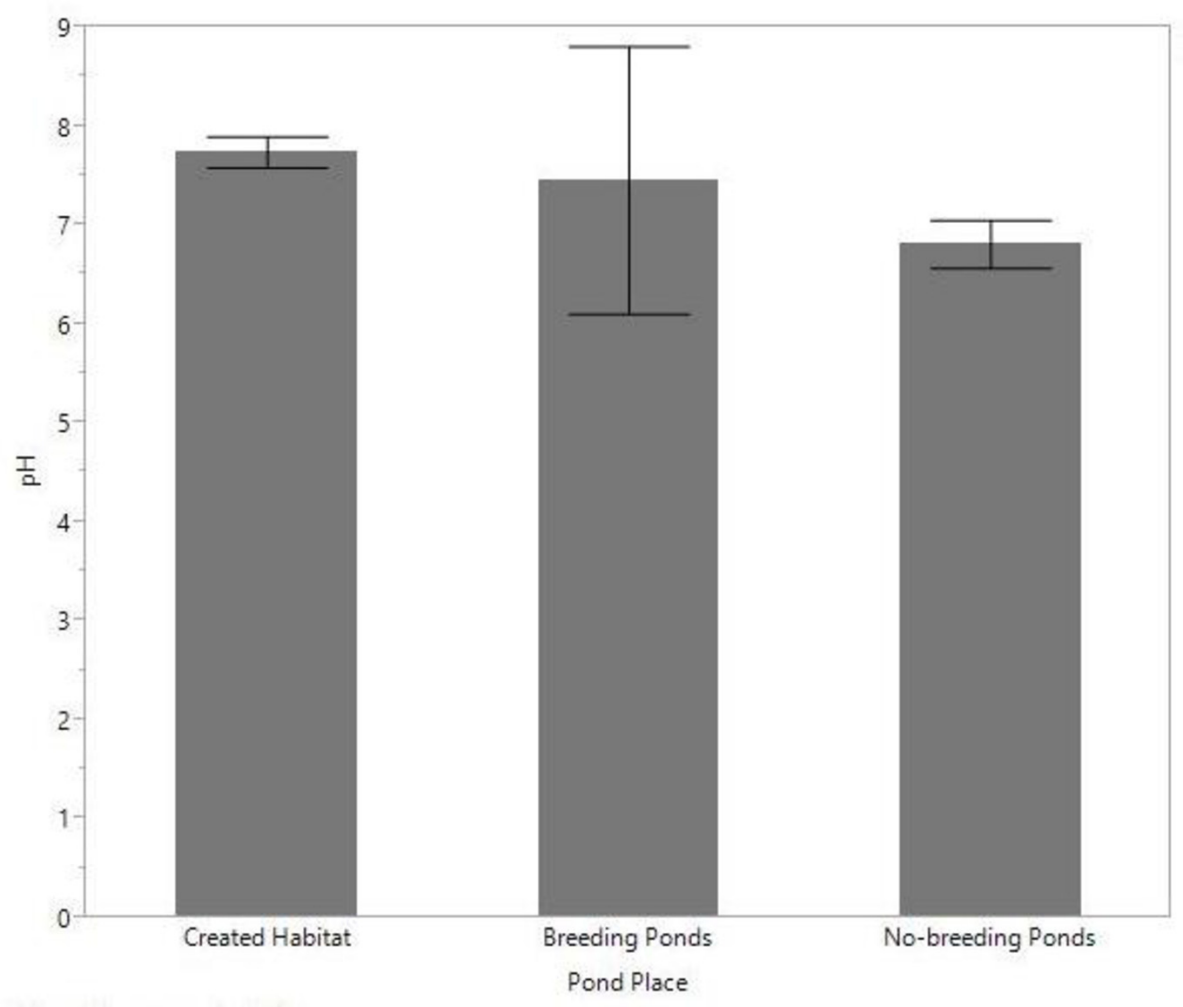
Mean pond pH levels across each of the pond places. Error bars represent 95% confidence intervals.

Ponds within the created habitat had significantly lower conductivity (ms/cm) (mean ± SD = 92.5 ± 374.2) compared to both breeding (1795 ± 1556.8, *Z* = 4.4, *P* < 0.001) and non-breeding ponds (3195 ± 8473, *Z* = 11.2, *P* < 0.001) (Fig 5), and significantly higher pH (7.7 ± 0.7) than non-breeding ponds (6.8 ± 1.4, *Z* = 5.5, *P* < 0.001), but not breeding ponds (7.4 ± 1.3, *Z* = 1.4, *P* = 0.16) (Fig 6). These water quality parameters did not differ between breeding and non-breeding ponds. The mean ± SD salinity levels (in ppt) did not vary between the created habitat (0.99 ± 4.6), breeding ponds (0.80 ± 0.59), and non-breeding ponds (1.8 ± 5.5) (*F* = 0.9, *P* = 0.4), nor did percent of dissolved oxygen (*F* = 0.71, *P* = 0.49) (created habitat = 62.4 ± 40.3, breeding ponds = 46.6 ± 35.4, non-breeding ponds = 68.7 ± 38.0), or total dissolved solids (mg/L) (*F* = 0.42, *P* = 0.65) (created habitat = 1192 ± 3934, breeding ponds = 385 ± 401.3, non-breeding ponds = 1444.6 ± 2582.8).

#### Comparisons within the created habitat

The vegetation structure, water quality, and aquatic invertebrate abundance and diversity were very similar between the fenced and unfenced sections of the created habitat. One-way ANOVAs for all of the vegetation variables mentioned above gave a result of P > 0.05, apart from riparian rock (F = 6.8, P = 0.01), and shoreline *T*. *orientalis* (F = 6.2, P = 0.01) and *P*. *australis* (F = 5.2, P = 0.02). No statistical difference was found between the fenced and unfenced section of the created habitat for any of the water quality variables tested, nor for the diversity or abundance of aquatic fauna collected in dip-netting surveys.

### Threats

#### Mosquitofish

*Gambusia holbrooki* colonised a permanent pond in the created habitat in November 2011, and continued to inhabit that pond for the rest of the survey years. *Gambusia holbrooki* also colonised two other created ponds, but did not persist. In the natural habitat, *G*. *holbrooki* were found in 46 ponds, but were only detected in 17 ponds every survey. The proportion of ponds that contained *G*. *holbrooki* at least once during the multiple survey periods varied significantly between pond place (*x*^*2*^ = 20.0, *P* < 0.001) with *G*. *holbrooki* captured in 83.6% of non-breeding ponds compared to 27.3% of created habitat ponds, and 28.6% of breeding ponds. The observed number of ponds that were occupied by *G*. *holbrooki* also varied from the expected values, with the created habitat containing three ponds that held *G*. *holbrooki* as opposed to the expected eight; two breeding ponds contained *G*. *holbrooki* as opposed to the expected five; and 46 non-breeding ponds contained *G*. *holbrooki* as opposed to the expected 38.

#### Bd screening

**Fig 7:**
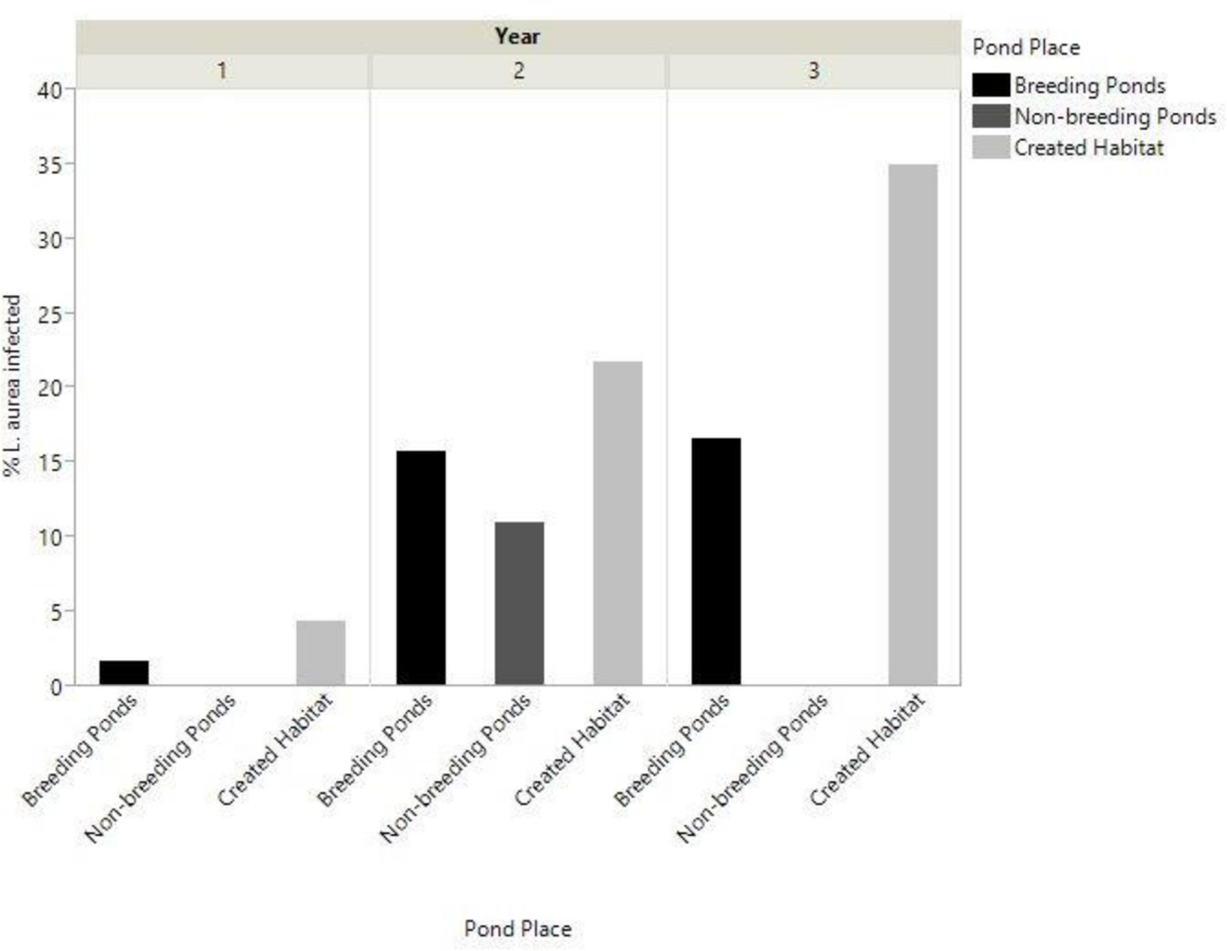
Comparison of Bd prevalence between sites. Percentage of *L*. *aurea* that tested positive for Bd across each site for the three survey years.

During the three survey years, 404 swabs were processed, of these 290 swabs represented individual frogs and were included in analysis. Ninety two swabbed frogs were from the breeding pond, 69 from the non-breeding pond, and 129 from the created habitat. The prevalence of Bd infected frogs across all years was 20% at the created habitat, significantly greater than the prevalence of infection at breeding ponds (7.5%), and non-breeding ponds (2.7%) (*x*^2^ = 16.7, *P* < 0.001). The prevalence of Bd fluctuated significantly between years (*x*^2^ = 14.2, *P* < 0.001), with an increase of infections in the second year at all sites, and a further increase in infection at the created habitat in the third year (Fig 7). For *L*. *aurea* that tested positive to Bd, there was no difference in infection severity between sites (*F* = 0.7, *P* = 0.5).

## Discussion

Several factors suggest that the created habitat should have been suitable for frog reproduction; released tadpoles survived through to post-metamorphosis and reproductive age, other frog species spawned in all of the created ponds, and although some water quality variables differed from other ponds on the island they were not outside of tolerance levels for *L*. *aurea* tadpoles [42]. There were however, less reproductively available male and female *L*. *aurea* per pond in the second and third survey years which may have influenced breeding behaviour. However, the greatest differences between the created habitat and natural ponds, particularly breeding ponds, were seen in vegetation composition in each of the three sampled zones, invertebrate abundance, and the prevalence of the pathogen Bd. Lower vegetation diversity may lead to lower invertebrate abundance and therefore lower food availability. Additionally, energy from food may be used in an immune response against pathogens rather than being directed to the growth of gonads or secondary sexual traits. Therefore these two factors (nutrition and Bd) may physiologically impact the frog’s ability to reproduce, and it is these physiological impacts, rather than habitat choice, that may have had the biggest impact on breeding in the created habitat.

### Demography

*Litoria aurea* can reach reproductive maturity within their first year, with reports of males breeding at three months post-metamorphosis, although it is more typical for males to breed at one year, and females at two [24, 37]. Therefore, it could be expected that breeding should have occurred at least by the third year post release of tadpoles. Male *L*. *aurea* have a loud, conspicuous mating call, and usually aggregate to form choruses in the mating season [43, 44]. This behaviour can lead to conspecific attraction of males towards other males to form a chorus, or females toward the chorusing males [44]. As calling was significantly more frequent in breeding ponds compared to the created habitat, it is therefore possible that conspecific attraction to wild frogs drew reproductively active frogs out of the unfenced section of the created habitat. During our third survey season we captured juveniles in the created habitat whose genetic results suggest that at least one parent was a released frog (Valdez 2015 unpublished data). Due to the high frequency of our tadpole surveys, and the fact that juvenile *L*. *aurea* often disperse from their natal pond [43], it is more probable that a breeding event occurred at a nearby pond, rather than occurred undetected at the created habitat. Whilst this result does not reflect our aim of obtaining breeding within the created habitat, it does highlight the importance of placing reintroduction programs within close enough proximity to a metapopulation of conspecifics so that these sub-populations can support each other [45]. This is likely to be highly relevant for *L*. *aurea* as both adults [46] and juveniles [43] have been shown to disperse.

Dispersal however, does not explain the lack of breeding within the fenced area where frogs were contained. Female frogs are often stimulated into reproductive readiness by male calling [47], therefore the lack of calling at the created habitat may have failed to trigger female physiological responses of increased estrogen levels and egg development, and subsequent eagerness to breed. Male frogs have also been found to increase androgen levels in response to conspecific calls [48], which can lead to increased testes mass [49]. Therefore a lack of calling at the trial site likely contributed to a lack of breeding. Whilst future created habitats or reintroduction programs would ideally aim to support natural frog calling, it is possible that the inclusion of artificial call-playback methods might stimulate calling behaviour, and subsequent breeding behaviour, and could be included in future management efforts [44].

Many frog species avoid ovipositing in ponds that contain heterospecific tadpoles to avoid increased competition or predation of their offspring at the larval stage [50, 51]. Breeding by heterospecific frog species occurred at least once in each of the created habitat ponds, but was unlikely to be why *L*. *aurea* did not breed at this site because the abundance of heterospecific tadpoles was equally high in breeding ponds, and even higher in breeding ponds than non-breeding ponds. These results suggest that the presence of heterospecific tadpoles has a negligible effect on *L*. *aurea* when selecting an oviposition site and pond, and that co-occurring frogs may require similar habitat traits when selecting breeding ponds. These results concur with previous research showing that *L*. *aurea* are more likely to breed in ponds containing a greater diversity of heterospecific amphibians [52].

### Habitat

Due to the placement of the created habitat in *Kikuyu* grass pasture, the created habitat had lower vegetation diversity and contained far fewer trees, bushes, reeds and sedges in the riparian and shoreline zones compared to the natural ponds. Previous studies have found that the presence of mid-sized trees and bushes positively correlated with the presence of *L*. *aurea* in these zones [29, 53]. Additionally, wetland studies have shown that terrestrial buffer zones around water bodies are extremely important for numerous amphibian species as they provide shelter from predators and desiccation when frogs are foraging or moving between sites [54, 55, 56]. However, *L*. *aurea* is considered a habitat generalist [29, 37] and several other studies of this species’ habitat, have not found a link to a particular vegetation type or community structure [43, 57].

Vegetation diversity is often important for invertebrate abundance and diversity [58], and can thus affect food resource availability for frogs. Indeed, we found that the created habitat had significantly lower invertebrate abundance and diversity compared to a breeding pond with higher vegetation diversity. Invertebrate diversity and biomass positively correlated with *L*. *aurea* growth rates in Sydney Olympic Park [59]. Food and nutrient availability also has a large impact on an animal’s reproductive output [11, 12]. For example, only female black bears, *Ursus americanus*, inhabiting areas with abundant carbohydrate food availability, were found to successfully rear offspring [11]. This link between energy intake and reproduction may be particularly relevant for frogs as reproduction for either sex is energetically expensive. Male calling to attract females, is one of the most energetically costly, male breeding behaviours in the animal kingdom [60]. Similarly, female frogs can produce large volumes of eggs, with *L*. *aurea* producing upwards of 3000 eggs per season, contributing to more than a quarter of their body weight [61]. As the vegetation structure and aquatic fauna was similar both inside and outside the fenced area of the created habitat, a lack of food resources may help explain the lack of calling behaviour and breeding across both sections of the created habitat.

In addition to terrestrial habitat, the aquatic habitat inside ponds also contained a lower percentage of plants, and a higher percentage of open water compared to breeding ponds. Because *L*. *aurea* attach their egg mass to aquatic vegetation [61], they may use the presence of emergent vegetation when selecting an oviposition site and pond. Therefore the lack of emergent vegetation may have deterred *L*. *aurea* from selecting these ponds for breeding and promoted dispersal to other more suitable areas outside of the created habitat.

Pond size also varied significantly between the created habitat and pre-existing ponds. On average, created ponds were smaller than natural breeding and non-breeding ponds on the island, and dried rapidly during dry conditions—although water levels were manually increased to prevent complete drying within the site. We suspect that the rapid water level decline, rather than pond size, deterred *L*. *aurea* from selecting the created ponds for breeding because: two natural ponds selected for breeding in this study were smaller than those within the created habitat; pond size did not correlate with *L*. *aurea* breeding patterns in previous studies [52]; and *L*. *aurea* tadpoles are poorly adapted to developing in waterbodies with decreasing water levels [27]. However, *L*. *aurea* have been observed dispersing to ephemeral waterbodies to breed after large rain events [46], suggesting that pond hydrology is not the only factor limiting breeding within the created habitat.

### Threats

#### Fish

Whilst the invasive mosquitofish is an effective predator of tadpoles [62, 63, 64], and has been implicated in the decline of several amphibian species [65, 66], including *L*. *aurea* [67], several studies have shown that adult *L*. *aurea* appear to be naïve to the predatory threat of *G*. *holbrooki* on tadpoles, and do not avoid the fish when choosing an oviposition site [53], (Klop-Toker unpublished data). We consider it unlikely that the presence of these fish would have deterred *L*. *aurea* from breeding within the created habitat due to this apparent naiveté and because only one pond within the created habitat contained *G*. *holbrooki* for an extended period of time. Our frequent sampling, plus the observation of tadpoles of co-occurring frog species in the *G*. *holbrooki*-occupied ponds, makes us confident that we did not miss a breeding event within the created habitat due to *G*. *holbrooki* predation of spawn or tadpoles. However, the presence of *G*. *holbrooki* may have reduced the detection probability of tadpoles in natural ponds through predation or reduced tadpole activity [68], potentially resulting in missed breeding events and false negatives within the non-breeding pond category, and lower numbers of co-occurring tadpoles in non-breeding ponds compared to breeding ponds. Whilst this fish may have had minimal impact on *L*. *aurea* breeding in our created habitat, it is important to note that due to this fish’s predatory ability, the exclusion of *G*. *holbrooki* from created ponds or re-introduction sites should be maintained for the benefit of progeny survival when breeding does take place.

#### Bd infection

The amphibian fungal disease chytridiomycosis, caused by the pathogen *Batrachochytrium dendrobatidis* (Bd), can be lethal to hundreds of amphibian species [69, 70], including *L*. *aurea*. Bd is implicated as a major driver of *L*. *aurea’s* decline [24] and has been linked to the failure of previous *L*. *aurea* breed and release programs due to disease-induced mortality [21, 71]. Therefore, swabbing for Bd was a routine part of our monitoring for this population. We were able to confirm that Bd caused a high mortality rate of *L*. *aurea*, particularly in the first winter, which contributed to the lowered abundance (Valdez 2015 unpublished data). We also found that *L*. *aurea* in the created habitat had significantly higher prevalence rates compared to *L*. *aurea* in both the breeding and non-breeding ponds.

Pathogen mediated reductions in host reproductive output have been observed in numerous organisms [72, 73, 74], with mechanisms for this pattern ranging from reduced competitive fitness of infected adults [72], adaptive avoidance of infected individuals by conspecifics [75], or physiological changes resulting in lower gonad mass [74, 76]. A recent study investigating the effects of Bd infection on gonad mass in *L*. *aurea*, discovered that Bd infection significantly reduced male testes mass and fat stores, and this reduction in teste size was maintained months after frogs had been cleared of infection [76]. Lowered testes mass is commonly correlated with lower testosterone levels [49, 77] and lower sperm quantity [78, 79], thereby affecting competitiveness to find a mate [80] and fertilisation rates, respectively. Furthermore, a reduction in male fat stores may have implications for the calling ability of males, as this activity is energetically costly [60], and male frogs may forgo foraging in order to call [81], essentially relying on these fat stores to fuel mate attraction. Therefore, the high severity and prevalence of this disease at the created habitat may have substantially reduced breeding behaviour and physiology of *L*. *aurea* at the created habitat. Why Bd was higher at the created habitat is a subject for further investigation, and several factors such as nutrition, host age, and density may have contributed.

## Conclusion

The continued presence of *L*. *aurea* at the created habitat is a positive sign that *L*. *aurea* can persist in a created habitat. But the lack of breeding highlights the importance of habitat creation that facilitates all stages of a target-species’ life cycle. Due to intensive monitoring, we were able to investigate several factors that may have contributed to the absence of *L*. *aurea* breeding at the created habitat. As vegetation affects invertebrate abundance, and as nutritional up-take and disease both affect reproductive behaviour and physiology, it is likely that the combination of these factors was to blame for the absence of breeding. Fortunately, because this created habitat was used as a trial to inform larger-scale habitat creation, we have the opportunity to build upon this knowledge and integrate what we’ve learnt into an improved program. Future programs may benefit by the creation of ponds with more permanent hydro-periods and the placement of created habitat in close proximity to an extant population, planting a variety of plant species at each of the wetland zones (riparian, shoreline, and emergent) which may encourage oviposition and increase invertebrate abundance. The addition of plants that are known to attract invertebrates might also provide additional nutrition for reproduction and immune function. Recommendations to reduce Bd in the site should also be incorporated, and trialling artificial call playback may be worthwhile for stimulating breeding hormones and behaviour. This study exemplifies how monitoring and scientific analysis of a created habitat or reintroduction program can not only improve our understanding of the target species and ecological system, but also greatly improve existing, or initiate new adaptive management strategies.

## Supporting Information

S1 Fig Example of a breeding pond on Kooragang Island. Yellow line represents 50 m. Source image from Google Earth 2016.
(JPG)

S2 Fig Example of a breeding pond on Kooragang Island. Yellow line represents 50 m. Source image from Google Earth 2016.
(JPG)

S3 Fig Example of a non-breeding pond on Kooragang Island. Yellow line represents 50 m. Source image from Google Earth 2016.
(JPG)

S4 Fig Example of a non-breeding pond on Kooragang Island. Yellow line represents 50 m. Source image from Google Earth 2016.
(JPG)

S5 Fig File of Supporting Dataset. (XLSX)
