# Supplementary figures and images for: We Made Your Bed, Why Won’t You Lie in It? Food Availability and Disease May Affect Reproductive Output of Reintroduced Frogs
Source: PLoS One. 2016 Jul 27;11(7):e0159143. doi: 10.1371/journal.pone.0159143 (PMC4963099; doi:10.1371/journal.pone.0159143)

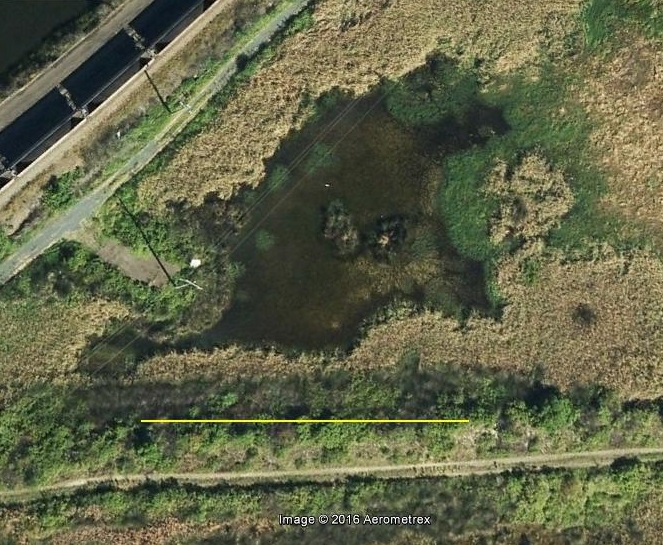

Supplement: S1 Fig — Yellow line represents 50 m. Source image from Google Earth 2016. (JPG) [file pone.0159143.s001.jpg]

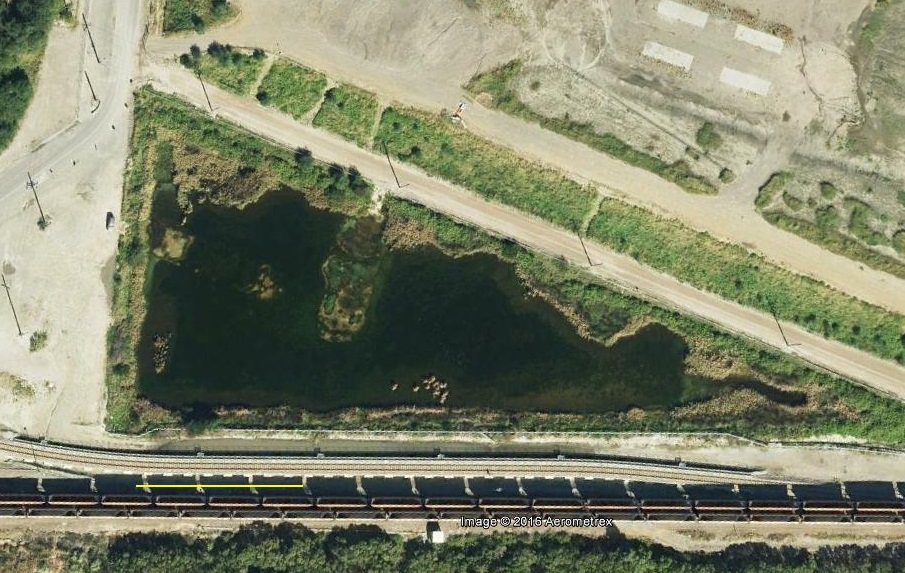

Supplement: S2 Fig — Yellow line represents 50 m. Source image from Google Earth 2016. (JPG) [file pone.0159143.s002.jpg]

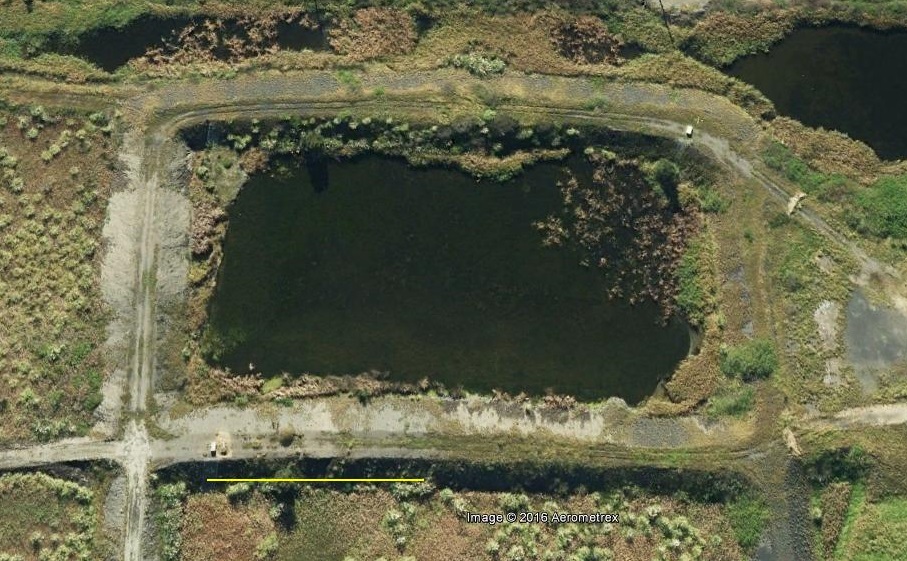

Supplement: S3 Fig — Yellow line represents 50 m. Source image from Google Earth 2016. (JPG) [file pone.0159143.s003.jpg]

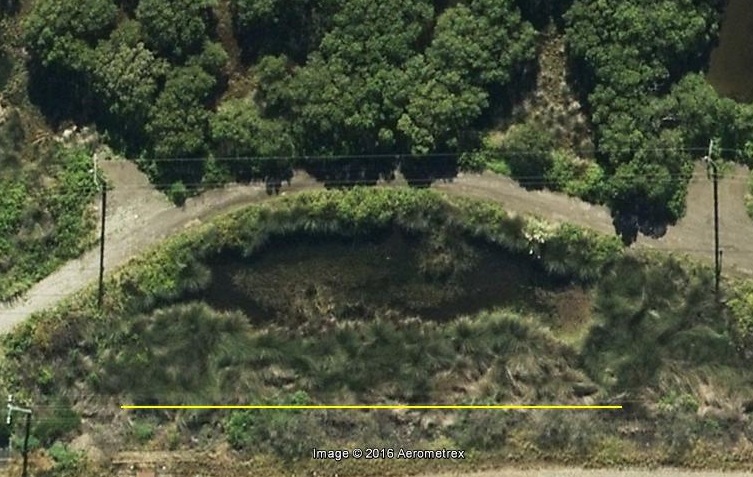

Supplement: S4 Fig — Yellow line represents 50 m. Source image from Google Earth 2016. (JPG) [file pone.0159143.s004.jpg]
